# PPAR agonists as effective adjuvants for COVID-19 vaccines, by modifying immunogenetics: a review of literature

**DOI:** 10.1186/s43141-021-00179-2

**Published:** 2021-05-31

**Authors:** Antoine Fakhry AbdelMassih, Rahma Menshawey, Jumana H. Ismail, Reem J. Husseiny, Yousef M. Husseiny, Shenoda Yacoub, Aya Kamel, Rafeef Hozaien, Elaria Yacoub, Esraa Menshawey, Abanoub Abdelmalek, Ahmed Abouelazaem, Ahmed Elhatw, Ahmed Aboelmaaty, Alaaelrahman Shahib, Amany Mansour, Aya Kamal, Basant Mohamed, Bemen Atif, Beshoy Ghabreal, Catherine Abdelmalak, David Ibrahim, Ebtesam Elsaify, Farah Magdy, Farid G. Hanna, Hadeer Hafez, Hafsa Dahir, Kerlos Merhom, Maram Ahmed, Mariam Bishara, Mina Tawfik, Mina Youssef, Mohamed El Sharnouby, Mourad Hamouda, Musheera Ammar, Nada Ali, Nada Daniel, Nadine El-Husseiny, Noha Abdelraouf, Nuran K. Abdelhameed, Radwa Ahmed, Radwa Othman, Rahma Mohamadein, Rana Allam, Rana Elgendy, Rana Shebl, Saged Elsherbiney, Sarah Fouad, Sara Emel, Sara Owais, Sarah Hetta, Samah El-Saman, Shaimaa Abdelalim, Sherin Galal, Yara Asar, Yara Osman, Yasmeen Khalaf, Youstina Aziz, Yousra Khafagy, Nervana Gamal, Biagio Castaldi

**Affiliations:** 1 Pediatric Cardiology Unit, Pediatrics’ Department, Cairo University Children Hospital, Faculty of Medicine, Cairo University, Kasr Al Ainy Street, Cairo, 12411 Egypt; 2 Pediatric Cardio-Oncology Department, Children Cancer Hospital of Egypt (57357), Cairo, Egypt; 3 Research Accessibility Team, Student and Internship research program, Faculty of Medicine, Cairo University, Giza, Egypt; 4 Pulmonology Department, Faculty of Medicine, Cairo University, Giza, Egypt; 5 Research Accessibility Team, Student and Internship research program, Faculty of Medicine, New Giza University, 6th of October City, Egypt; 6 University at Buffalo School of Medicine and Biomedical, Buffalo, USA; 7 Research Accessibility Team, Student and Internship research program, Faculty of Medicine, Ain Shams University, Cairo, Egypt; 8 Faculty of Dentistry, Cairo University, Giza, Egypt; 9 Pixagon graphic design Agency, Cairo, Egypt; 10 Research Accessibility Team, Student and Internship research program, Faculty of Medicine, 6th October University, 6th of October City, Egypt; 11 Padova University, Padua, Italy

**Keywords:** COVID-19 vaccine, PPAR, Immunologic memory

## Abstract

**Background:**

Several coronavirus vaccine have been fast-tracked to halt the pandemic, the usage of immune adjuvants that can boost immunological memory has come up to the surface. This is particularly of importance in view of the rates of failure of seroconversion and re-infection after COVID-19 infection, which could make the vaccine role and response debatable. Peroxisome proliferator-activated receptors (PPARs) have an established immune-modulatory role, but their effects as adjuvants to vaccination have not been explored to date.

**Main body of the abstract:**

It is increasingly recognized that PPAR agonists can upregulate the levels of anti-apoptotic factors such as MCL-1. Such effect can improve the results of vaccination by enhancing the longevity of long-lived plasma cells (LLPCs). The interaction between PPAR agonists and the immune system does not halt here, as T cell memory is also stimulated through enhanced T regulatory cells, antagonizing PD-L1 and switching the metabolism of T cells to fatty acid oxidation, which has a remarkable effect on the persistence of T memory cells. What is even of a more significant value is the effect of PPAR gamma on ensuring a profound secretion of antibodies upon re-exposure to the offending antigen through upregulating lipoxin B4, therefore potentially assisting the vaccine response and deterring re-infection.

**Short conclusion:**

In view of the above, we suggest the use of PPAR as adjuvants to vaccines in general especially the emerging COVID-19 vaccine due to their role in enhancing immunologic memory through DNA-dependent mechanisms.

## Background

The ongoing humanitarian crisis caused by COVID-19 which started in Wuhan has brought the world to a standstill [1, 2]. As of yet, COVID-19 cases have surpassed 160 million individuals, causing more than 3 million deaths, and counting [3].

Several widely anticipated vaccines targeting COVID are underway, the main constitutions of which are either recombinant viral-vectored, mRNA-based, or protein subunit vaccines. SARS-COV-2 has various structural proteins. Its S protein, in particular, generates antibodies that were found to be neutralizing to the virus. Consequently, all COVID-19 vaccines incorporate at least a portion of S protein [4]. S protein subunit vaccines mainly work by inducing CD4+TH cell and antibody response; however, they had a weaker CD8+T cell response [5].

They were found to require adjuvant and repeated doses to effectively stimulate high titers of neutralizing antibodies to minimize the possibility of antibody-dependent enhancement of disease (ADE) [6]. mRNA-based vaccines, complexed with lipid nanoparticle carriers, induce strong antibody response targeting S proteins as well as CD4+ and CD8+ T cell response. However, the magnitude and duration of these immune responses are still in question. Observing the development of B or T cell memory using next-generation sequencing, as well as the rate of seroconversion and lastly the re-infection rates and severity, can provide useful insights on the extent of immunologic memory developed after COVID-19. These insights can give us an idea about the potential longevity of protection provided by the vaccines under-development and how to improve it.

The SARS-CoV infection is thought to disrupt multiple aspects of immunity. In particular, Liao and colleagues emphasized the lack of evidence for B cell memory generated in severe patients during the early recovery period. This might potentially render them susceptible to COVID-19 re-infection and decreases the likelihood of developing long-lasting immunologic memory [7].

Indeed, re-infection from COVID-19 has been a disappointing matter that has turned around the approach of post-infection cases’ follow-up. A study reported that 11% of recovered COVID-19 Chinese patients were re-infected although they were asymptomatic [8]. Moreover, surveillance after patients discharged in Guangdong, China, reported that 9.5% got re-infected [9]. A systematic review conducted by Gidari et al. with a total of 82 papers thoroughly searched concluded that 1350 patients that were infected with COVID-19 had manifested with positive respiratory investigative criteria after recovery [10]. Another report by AbdelMassih and colleagues concluded that of the re-infected cases especially those who failed to seroconvert after the initial infection developed more severe manifestations in the re-infection episode.

Despite a promising recent report by Lumley et al. who showed that seroconversion conveys immunity and shields patients from severe re-infection, however, antibodies have been found to wain within 2 months after infection, suggesting shortened protection. Another challenge to long-lasting immunity from COVID-19 is the non-negligible percentage of patients who fail to seroconvert after initial infection, which is approximately 10–12% in most of the series exploring antibody status in affected patients [11].

Peroxisome proliferator-activated receptors (PPARs) are members of the nuclear hormone receptor superfamily of which there are three isoforms: PPARα, PPARβ/δ, and PPARγ. It increasingly recognized that members of the nuclear-receptor superfamily have significant regulatory effects on inflammatory processes [12] and thus PPAR agonists and antagonists have been implemented in multiple clinical trials for their immunomodulatory role. Distinctively, a study highlighted using a mouse model that PPARγ is crucial for optimal humoral immune responses. The study showed that mice lacking PPARγ expression in B cells had decreased proliferation and IgG production in vitro, as well as low levels of circulating antigen-specific antibodies during a primary response. This was also reflected in the immune memory response where PPARγ-deficient mice had a form of impairment of said immunity with low numbers of antigen-experienced antibody-secreting cells. The results showed the importance of PPARγ expression in B cells during both the primary and secondary immune response [13]. Another study showcased that physiological doses of PPARγ ligands alone or in combination with other ligands accelerate the differentiation of B cells into plasma cells resulting in the heightened synthesis of immunoglobulins. The literature supports that the PPARγ activation pathway can be utilized to boost the humoral immune response [13]. Despite the increasing body of evidence that PPAR can have an immunomodulatory role in the context of COVID-19 infection, its role in improving vaccine responses has not been explored to date.

These receptors all share in common a unified structure as follows: N-terminal ligand-independent transactivation domain, a DNA binding domain, and a C-terminal ligand-binding domain and ligand-dependent activation domain. Having both a DNA binding domain and a ligand-binding domain that can be stimulated by different types of agonists-drugs has made these molecules an important tool for the modification of DNA transcription in various cells to modify cell behavior towards a required therapeutic target. As mentioned earlier, one of the most important therapeutic effects that are being a point of focus in the recent years is their immunomodulatory role through interaction with nuclear DNA of various types of immune cells. However, despite thorough investigations in this context, none has focused on their role in improving vaccination responses, the so-called, vaccination adjuvant role. Several vaccination adjuvants have been tailored over years to be coupled to vaccines to enhance immune responses and seroconversion after vaccination [14]. The major adjuvant established for most of the available vaccines is aluminum. Its mode of action was based upon prolonging the interaction between the antigen and the immune system by delaying the degradation of the antigen provided by vaccination. This old strategy is being slowly replaced by a newer strategy, which is the stimulation and modification of immune cells’ behavior through stimulation of Toll-like receptors, crucial receptor of the innate and adaptive immune system to enhance their reactivity to the administered antigen. Most of the investigated molecules are either portions of double-stranded DNA (ds DNA) or small ligand proteins [15].

Immunological memory is the cornerstone of vaccine effectiveness; therefore, the competent regulation of memory cells’ expression will highly guarantee promising vaccine outcomes. With anticipation growing, for an answer for this unprecedented global pandemic, any potential vaccine alone might not be the end all be all, potentially needing to be fortified to ensure its potency. The augmented proliferation of memory B cells is proven to be in a direct correlation with the use of PPARγ ligands. Garcia-Bates et al. [14] have also demonstrated the influence of PPARγ ligands’ downregulation on T cell proliferation, which will furthermore boost the expression of IFN-γ and IL-12. The cytokines released in response to T cell functioning synergistically enhance the roles of B cells in combating current and foreseen re-infection.

Given this new strategy, and the potential of PPARs to modify and enhance the reactivity of immune cells through epigenetic and DNA-linked mechanisms, we hypothesize that PPARs can be proposed as potential and affordable adjuvants for vaccines. The aim of this work is to prove such hypothesis through a thorough review of the available literature of the effect of PPARs on B and T cell memory.

## Main text

### Prolonging B cell memory and improving secondary antibody response

B cells are an essential component of humoral immunity [16]. Immature B cells that are exiting the bone marrow acquire immunoglobulin (Ig)D, cluster of differentiation (CD)21, and CD22 on their surface [17]. Following antigen exposure, most of the B cells outside the gut-associated lymphoid tissue (GALT) which reside in the spleen and lymph nodes develop immunologic memory through four steps:

(1) Respond to T cell-dependent foreign antigens, (2) proliferate, (3) differentiate into long-lived plasma/memory B cells (secrete abundant amounts of antibodies), or (4) enter into the germinal center (GC) [18–20]. Therefore, the optimal humoral response depends on the formation of antigen-specific titers that are produced by non-proliferating long-lived plasma cells (LLPC), which are located within the bone marrow. The hallmark of LLPC is longevity. Only recently, are we beginning to understand the functions and mechanisms of survival of these cells even after years of antigen exposure. It should be noted that LLPCs are not intrinsically long-lived and require continuous signaling from their LLPC niche in order to survive. We are exploring in this section the signals needed to maintain the survival of LLPCs and their relationship with the PPAR pathway [21].

#### PPARγ induced upregulation of anti-apoptotic factors

Signals that are responsible for the upregulation of the anti-apoptotic factor Mcl-1, which is expressed on LLPC, are essential for LLPC survival [21].

BCL-2 family can be considered a tripartite apoptosis control system consisting of (1) a set of anti-apoptotic proteins and (2) two sets of pro-apoptotic proteins, both of which collaborate to determine the survival or death of the cell in different pathophysiological states: 1, 2, 3, and 4. BCL-2, BCL-XL, BCL-W, MCL-1, and A1/BFL-1 are five known anti-apoptotic members that are similar in four BCL-2 homologies (BH) domains [22].

Wu and colleagues succeeded in demonstrating the ability of Rosiglitazone, a PPAR γ agonist, in increasing cell viability and stability of the mitochondrial membrane. This effect was mediated through upregulation of the anti-apoptotic members of the BCL-2 family [23].

#### Metabolic fitness

Metabolic fitness is another essential component of LLPC longevity, which facilitates the diversion of glucose in order to generate pyruvate in a state of stress to facilitate long-term survival [21].

PPAR induces and orchestrates a switch from glucose to fatty acid utilization for energy production in hepatocytes. Notably, there is simultaneous activation of FAO by PPAR*α* and inhibition of glycolysis. PPAR*α* activation reduces PK expression and induces pyruvate dehydrogenase lipoamide kinase. This effect will generate pyruvate and will prevent pyruvate internalization into the mitochondria, thus providing a metabolic niche for the long-term survival of LLPCs [24].

#### Cellular partners of LLPCs

The LLPC niche is the third major component of LLPC survival. It is composed of other cellular partners such as dendritic and T regulatory cells, which promote survival signaling by:

1. The expression of ligands such as CD80/CD86 for CD28 and
2. Produce soluble and stromal factors, which contribute to LLPC longevity [21]

Wang and associates were able to study the effect of PPAR γ on Treg cells in a murine model of allergic rhinitis and reported that pioglitazone significantly increased the expression of Foxp3 mRNA as well as the population of Tregs [25].

In a study assessing the effects of PPAR γ agonists on dendritic cells, Szatmari et al. found that PPARγ orchestrates a transcriptional response, which results in the development of a DC subtype with (1) increased internalizing capacity, (2) efficient lipid presentation, and (3) an augmented potential to activate iNKT cells and LLPCs [26].

We can conclude from the above that both PPAR γ and α can increase the longevity of LLPCs and therefore improve the results of vaccination.

#### Improving secondary antibody response through PPAR

Another mechanism that can explain inter-personal variability to vaccines is the ability of LLPCs to produce an efficient secondary antibody response. This potential has been of special interest to Kim and colleagues. The latter analyzed the molecular basis of such potential and demonstrated that secondary antibody response is mediated by “proresolving mediators.” This family comprises a large group of endogenously produced lipids, of which lipoxin B4 particularly operates to stimulate memory B cells to secrete antibodies upon re-exposure to the offending antigen [27, 28].

Lipoxin B4 seems to act through several intertwined molecular mechanisms, namely retinoic acid pathway, COX-2, and Blimp-1. All of which including lipoxin B4 were proved to be upregulated by PPARγ.

#### PPARγ and evidence from vaccinations

A vaccine can generate specific long-lived plasma cells, memory B cells, and serum antibodies [29]. Although it is unclear as to how these elements are preserved over time, it should be noted that when judging the efficacy of a vaccine, we only measure serum antibodies, which are just one of the three components of immunological memory [29].

After primary immunization, long-lived plasma cells produce the remaining specific antibodies in the body, whereas boosters increase as a result of memory B cells [29]. There are two hypotheses regarding “serological memory”; the first suggests that long-lived plasma cells are sufficient to maintain memory, whereas the second theory suggests that serological memory is replenished by continuous stimulation of memory B cells [29]. Memory B cells, responsible for fast recall responses, have a limited life span and therefore suggest the need for cyclic re-stimulation to maintain memory against a particular pathogen [29].

Kye et al. [30], when assessing the effect of PPARγ on intranasal vaccination of *Streptococcus pneumoniae* in mice, found that serum antibody levels of mice injected with PPARγ antagonist (GW9662) before vaccination was markedly reduced in comparison to the mice that received the vaccination directly. This indicates that PPARγ is one of the most highly upregulated genes that induce long-term immunity via B cells [30]. After injection of a lethal dose of *S. pneumoniae* 12 weeks after vaccination, there was 100% survival rate, thus confirming the use of PPARγ and its agonists as a benefit in the setting of vaccination [30].

This data would further prove our hypothesis that the use of PPARγ agonists would promote the proliferation and differentiation of B cells in a synergistic manner, which would enhance the memory of B cells. In the setting of COVID-19 vaccination, the use of these substances will help to maintain serological memory against SARS-COV-2 viral particles may be through a “booster effect,” which has yet to be proven.

### Prolonging T cell memory with PPAR

PPAR-γ was originally identified as the molecular target for the thiazolidinedione (TZD) class of antidiabetic drugs [31]. Subsequent medical research indicated that PPAR-γ is highly expressed in secondary lymphoid organs and cells of the immune system and it has also been documented that PPAR-γ loss leads to enhanced proliferation of lymphocytes, which reflects the important role PPAR-γ plays in regulating immune responses [32].

Given the high impact on the inflammatory process of PPAR-γ activation, the use of PPAR-γ agonists has been suggested as one of the potential therapeutic compounds capable of treating cytokine storms that usually take place during severe viral influenza. For example, in 2009, Aldridge et al. showed that administration of pioglitazone to mice increased the rate of CD8+ T cells in infected lungs leading to decreased morbidity and mortality due to the influenza virus A [33]. A second study was carried out in 2010 which endorsed the therapeutic use of thiazolidinedione, demonstrating the effectiveness of rosiglitazone and pioglitazone in reducing the cytokine storm-induced inflammatory mechanism in the H1N1 influenza A virus in the mouse model [34]. One of the sites where PPAR is expressed is the lungs [35]. Studies have shown that mice deficient in PPARα have an increased inflammatory pulmonary response to lipopolysaccharide-induced inflammation [36]. As a result, the decrease in PPARα due to COVID-19 can potentially be the main factor in the cause of pulmonary inflammation and be involved in the pathogenesis of acute lung injury [35]. However, the role of PPAR in improving B or T cell-mediated immunologic memory, and its subsequent potential to improve vaccine responses, was not explored to date.

#### Stimulation of T cell memory through enhancing T regulatory cells.

Recent evidence shows that PPAR-γ plays an important role in regulating the plasticity of Th17 to iTreg multiple functional phenotypes. Additionally, PPARy is involved in T cell differentiation to Th17 or iTreg cells by upregulation of Th17 receptors and downregulation of iTreg cell receptors (FOXP3) in mice. Computational results, adoptive transfer studies, and co-transfer studies in mice supported the initial prediction that PPARy activation increases iTreg cell differentiation.

Moreover, there were more short-lived effector CD8+ T cells during the peak of the immune response in the spleens of mice lacking Treg, but the memory CD8+ T cell response was impaired. Therefore, Treg-dependent production of TGF-β led to an increased expression of CD103 on CD8+ T cells, providing a large pool of resident memory T cells to be maintained in the brain post-infection [37].

#### Upregulation of γδ TCR and its role in improving T cell survival

T cell diversity is an essential aspect of an effective immune system. Two entities of T cells have been identified, namely γδ and αβ T cells. Studies on murine models conclude that subpopulations of γδ T cells can develop long-lasting memory with protective properties in comparison with the conventional αβ T cells [38]. Study shows TCRγδ is relatively increased by around 5 folds via PPARβ stimulation [39].

#### Switching the metabolism of T cells to fatty acid oxidation

We have come to the understanding that PPARs’ physiological function is through their action as transcription factors, controlling the expression of specific target genes. PPAR alpha modulates the transcription of genes involved in beta-oxidative degradation of fatty acids while PPAR gamma acts on glucose homeostasis by helping the differentiation of immature adipocytes to mature adipocytes [40]. They are also related to the nuclear hormone receptor superfamily and control multiple physiological functions such as development, energy metabolism, cellular differentiation, and inflammation.

Lipid metabolism has a significant effect on T cells’ fate and function [41], and generation of CD8+ memory T cells requires metabolic reprogramming that is characterized by enhanced mitochondrial fatty acid oxidation (FAO) [42]. Le Menn et al. declare that CD8+ memory T cells were found to heavily rely on FAO to meet their metabolic demands, and therefore, the expression of carnitine palmitoyl-transferase 1a (CPT1a), which is the limiting enzyme of FAO, is proven to shift the differentiation of T cells towards the CD8+ subset [43]. Memory CD8^+^ and Treg cells, which rely on FAO [44], utilize FAO to support their development and long-term survival without the need to rely on extracellular fatty acids [45]. Previous studies demonstrating the association between FAO and the enhanced survival of CD8+ memory T cells have helped establish the link between FAO and cellular longevity in the immune system [42].

Moreover, FA derived from lipolysis can fuel oxidative phosphorylation (OXPHOS), and lipolysis is also required for the production of lipid signaling molecules, such as lipid ligands, to activate the peroxisome proliferator-activated receptor (PPAR) pathway [42]. Furthermore, fatty acid binding proteins (FABP) 4 and 5 control lipid uptake and metabolism and thus act as a regulator of tissue-resident memory CD8+ T cell function in the lung [46], and PPAR-γ regulates their expression [47].

Mothe-Satney et al. conducted a study that proved that stimulation of the peroxisome proliferator-activated receptor β (PPARβ) increases fatty acid oxidation in T cells. It showed that activation of PPAR-β supports fatty acid oxidation instead of aerobic glycolysis and this favors the increase in the long-surviving memory T cells. Moreover, it showed that PPAR-β controls the expression of genes related to fatty acid oxidation such as acetyl-CoA acyltransferase 2 (Acaa2), very long-chain acyl-CoA dehydrogenase (Acadvl), and Cpt1a [39].

PPAR-γ is essential for the development of preadipocytes to adipocytes [48]; although it inhibits the generation of effector cells with Th17 properties, it increases the generation of the memory cells as it depends on fatty acid metabolism rather than glycolysis. In addition, PPAR-γ directly binds on DNA and controls the expression of genes related to fatty acid uptake and oxidation [48], whereas PPAR-α regulates genes involved in oxidative degradation of fatty acids. Another study suggested that FAO was promoted by using PPAR-α agonist, fenofibrate, which improved CD8^+^ TIL’s ability and synergized with PD-1 blockade. This effect could have been linked with enhanced oxidative phosphorylation, and mitogenic ROS production from mitochondria is supported by similar studies in which treatment with PPAR activator bezafibrate combined with PD-1 blockade, but not alone, led to CD8^+^ T cell activation through mitochondrial expansion [49, 50]. Therefore, having an increased mitochondrial mass and enhanced sparse respiratory capacity allows memory T cells to rapidly respond to an antigen-mediated rechallenge [45].

#### Antagonizing PD-L which prevents T memory cells from developing

PPAR-ɑ agonist, through fatty acid catabolism, improves T cell lymphocyte functions and enhances the therapeutic effect of PD-1 blockade in melanoma. FAs are converted to acetyl-CoA that acetylate key enzymes within the TCA cycle, which increases GAPDH’s enzymatic activity and reduces its binding to the 3′UTR region of IFN-g mRNA, thus enhancing IFN-y production [51] and T cell effector functions.

CD8+ T lymphocyte exhaustion and loss of effector functions are signaled by high expression of PD-1 as it facilitates the CD8+ TILs’ metabolic switch within a Glc-poor tumor microenvironment (TME) [52]. Therefore, the efficacy of cell therapy in patients with cancers characterized by low glucose content can be enhanced by PPAR-ɑ agonist’s metabolic reprogramming of CD8+ T cells [49].

**Fig. 1:**
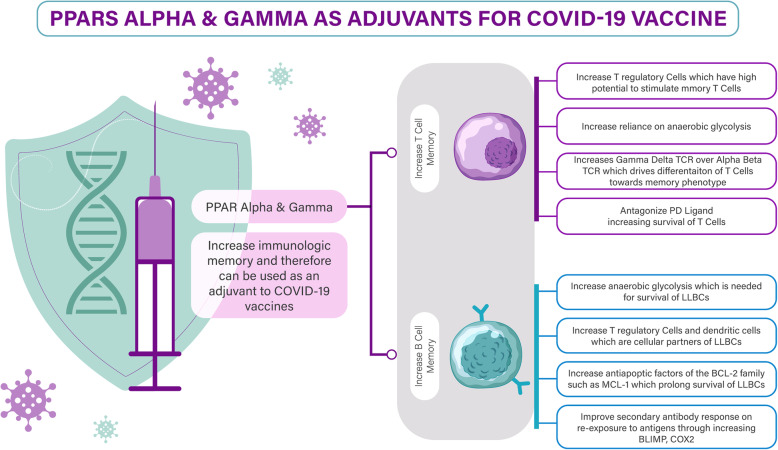
PPARs alpha and gamma as adjuvants for COVID-19 vaccine. Abbreviations: BCL B cell lymphoma protein, BLIMP B lymphocyte-induced maturation protein, COVID-19 coronavirus disease 2019, COX cyclo-oxygenase, MCL myeloid cell leukemia protein, LLBCs/LLPCs long-lasting B/plasma cells, PD programmed death, PPAR peroxisome proliferator-activated receptor, TCR T cell receptor

Furthermore, dendritic cells are essential for CD8+T cell activation, and during this process, PD-L and CD80 are expressed on their surface. Through a programming mechanism during this activation phase, differentiation of the effector and memory CD8+ T cells occurs, and during this programming, external stimuli as TCR signaling, co-stimulatory signaling, and cytokine signaling affect naïve CD8+ T cells [53]. In order to modulate the differentiation of effector and memory CD8+ T cells, PD-L1 signaling is integrated during CD8+ T cell activation. An investigation showed the effect of PD-L signaling during activation on the resulting antigen-specific CD8+ T cell memory population. An experiment was performed where PD-L1 blocking antibodies were given to mice before and after HSV-1 infection and mice were subjected to the virus later. The CD8+ T cell responses were evaluated after re-infection. The memory response in mice that activated the anti-HSV-1 CD8+ memory T cells in the absence of PD-L1 showed increased antigen-specific secretion of IFN-gamma and granzyme B [54, 55]. Figure 1 summarizes the mechanisms by which PPARs (alpha nad gamma) can act as adjuvants to the immune respone genertaed by curretly available COVID-19 vaccines.

## Conclusion

As vaccine effectiveness depends on immunological memory, this research throws light on how PPAR can be used to enhance both B memory and T memory cells through various mechanisms. Studies have shown that PPAR will improve the secondary antibody response of B cells through lipoxin B4, retinoic acid, COX-2, and Blimp-1; increase the lifespan of LLPC; and stimulate the upregulation of anti-apoptotic factors of the BCL-2 family. Moreover, studies have also shown that PPAR has an increased FAO on T cells stimulating the production of CD8+ memory T cells, an increase in TCRγδ by 5 folds approximately and antagonizing of PD-L, which prevents T memory cells from developing. Thus, we believe PPAR agonists would ensure a worthwhile vaccine response and prevent re-infection. We thereby urge working groups across the world who are testing the different types of COVID-19 vaccines to test the validity of this hypothesis through prospective trials.

## Data Availability

Not applicable.

## Abbreviations

BCL: B-cell lymphoma
BLIMP: B lymphocyte-induced maturation protein
CD: Cluster of differentiation
COVID-19: Coronavirus disease 2019
COX: Cyclo-oxygenase
DNA: Deoxyribonucleic acid
FABP: Fatty acid binding protein
FAO: Fatty acid oxidation
FOXP3: Forkhead box P3
Glc: Glucose
IFN: Interferon
IL: Interleukin
LLPC: Long-lasting plasma cell
MCL: Myeloid cell leukemia
NK: Natural killer
PD-L1: Programmed death ligand 1
PK: Pyruvate kinase
PPAR: Peroxisome proliferator-activated receptor
RNA: Ribonucleic acid
SARS-COV-2: Severe acute respiratory distress syndrome coronavirus 2
T reg: T regulatory
TCA: Tricarboxylic acid cycle
TCR: T cell receptor
TGF: Transforming growth factor
TH: T helper
TIL: Tumor infiltrated lymphocyte
Treg: T regulatory cell
UTR: Untranslated region
